# Degradation of Herbicides in the Tropical Marine Environment: Influence of Light and Sediment

**DOI:** 10.1371/journal.pone.0165890

**Published:** 2016-11-02

**Authors:** Philip Mercurio, Jochen F. Mueller, Geoff Eaglesham, Jake O'Brien, Florita Flores, Andrew P. Negri

**Affiliations:** 1 Australian Institute of Marine Science, Townsville, Queensland, Australia; 2 The University of Queensland, National Research Centre for Environmental Toxicology, Coopers Plains, Queensland, Australia; University of Illinois at Urbana-Champaign, UNITED STATES

## Abstract

Widespread contamination of nearshore marine systems, including the Great Barrier Reef (GBR) lagoon, with agricultural herbicides has long been recognised. The fate of these contaminants in the marine environment is poorly understood but the detection of photosystem II (PSII) herbicides in the GBR year-round suggests very slow degradation rates. Here, we evaluated the persistence of a range of commonly detected herbicides in marine water under field-relevant concentrations and conditions. Twelve-month degradation experiments were conducted in large open tanks, under different light scenarios and in the presence and absence of natural sediments. All PSII herbicides were persistent under control conditions (dark, no sediments) with half-lives of 300 d for atrazine, 499 d diuron, 1994 d hexazinone, 1766 d tebuthiuron, while the non-PSII herbicides were less persistent at 147 d for metolachlor and 59 d for 2,4-D. The degradation of herbicides was 2–10 fold more rapid in the presence of a diurnal light cycle and coastal sediments; apart from 2,4-D which degraded more slowly in the presence of light. Despite the more rapid degradation observed for most herbicides in the presence of light and sediments, the half-lives remained > 100 d for the PS II herbicides. The effects of light and sediments on herbicide persistence were likely due to their influence on microbial community composition and its ability to utilise the herbicides as a carbon source. These results help explain the year-round presence of PSII herbicides in marine systems, including the GBR, but more research on the transport, degradation and toxicity on a wider range of pesticides and their transformation products is needed to improve their regulation in sensitive environments.

## Introduction

Pesticides play an integral role in global food production; however, some have long persistence in the environment and are toxic to non-target species [1]. Chronic pesticide exposure has contributed to the decline of water quality in the Great Barrier Reef (GBR) region where intensive agricultural practices occur adjacent to sensitive marine habitats [2]. It is estimated that up to 30 tonnes photosystem II (PSII) herbicides are transported into the GBR lagoon annually [3] and concentrations higher than 10 μg l^-1^ can be detected in the receiving waters [4]. PSII herbicide concentrations in the GBR lagoon are generally lower but have exceeded current water quality guidelines [5, 6] and can affect photosynthesis and growth in sensitive marine organisms, such as seagrass [7, 8]. The five most commonly detected PSII herbicides, diuron, atrazine, hexazinone, tebuthiuron and ametryn, have been designated “priority herbicides” by management agencies and are monitored and managed as part of an overall GBR management and protection plan [9]. Although most extensively studied in the catchments and the lagoon of the GBR, PSII herbicides are present in nearshore marine systems across the world [10–14].

PSII herbicides are detected at higher concentrations during flood plumes which enter the GBR lagoon over the summer monsoon period [15, 16], but these contaminants are also found in low concentrations year round [17, 18] suggesting long environmental persistence. Evidence of degradation in the environment is shown by the detection of the breakdown products of atrazine and diuron in the GBR region [15, 16, 19] sometimes reaching concentrations over 2 μg l^-1^ at highly contaminated sites [20]. The persistence of contaminants in the environment is governed by the rates of multiple processes including hydrolysis, light/ UV driven photolysis and microbial degradation (metabolism) [21–23]. Microbial degradation is considered the dominant route of degradation for most PSII herbicides in aquatic systems [22, 24, 25] and our recent standard flask study indicates very slow degradation of diuron, atrazine, hexazinone and tebuthiuron in seawater, with evidence of both hydrolysis and microbial metabolism of these herbicides [26].

There is a relatively large number of *in situ* and laboratory studies on the persistence of PSII herbicides in freshwater aquatic systems (see [27]); however, few laboratory studies have examined persistence in seawater (summarised in [26]). The long seawater persistence of PSII herbicides observed in standard flask studies (half-lives >500 d) [26] indicates slower degradation than in freshwater, and may be influenced by a range of factors including salinity, pH and alternative organic carbon food sources and concentrations of other nutrients, which in turn affect microbial degradation and hydrolysis. Most previous studies on PSII herbicide persistence in seawater were not conducted for long enough to calculate reliable estimates of half-lives and several of these studies applied very high initial concentrations of herbicides (e.g. 5000 μg l^-1^) which may affect degradation rates by artificially influencing natural microbial communities and/or may be toxic to some components of the communities [26]. Our previous degradation studies applied the most natural conditions practical in a standard flask environment [26, 28], including low contaminant concentrations (~10 μg l^-1^), native microbial communities, no artificial nutrients and tests were conducted over 12 months under different light and temperature conditions [26, 29]. The application of low light increased the degradation rates of diuron, tebuthiuron and the non-PSII herbicide glyphosate, and slowed the degradation of the non-PSII herbicide 2,4-D [26, 29]. An increase in temperature from 25°C to 31°C increased degradation of diuron, hexazinone, tebuthiuron, 2,4-D and glyphosate and slowed the degradation of atrazine. While providing some of the first reliable standard flask persistence data in marine systems for several herbicides under highly controlled conditions, these studies highlighted the strong influence on persistence of field-relevant factors, including light and temperature [26, 29].

The potential environmental risks posed by pesticides can only be assessed when their fate in the environment, including potential degradation rates under field-relevant conditions are understood [1]. All previous studies testing the persistence of herbicides in seawater have been performed in standard flask tests in the absence of marine sediments and variable sunlight (summarised in [26]). Although useful for less persistent contaminants and for comparison of persistence across standard conditions, the rates of degradation may be unrealistic in short standard flask tests due to the absence of more natural microbial communities and conditions associated with open systems and coastal sediments. In order to determine the persistence of commonly detected herbicides under more natural conditions we conducted a year-long degradation experiment on commonly detected herbicides in a series of replicate open tanks. The experiment was not designed to assess degradation pathways, rather we tested the effects of natural parameters which might affect persistence, including natural light intensities and the presence of coastal sediments in some treatments. Therefore, the present study was designed to deliver more ecologically relevant persistence data for inclusion by regulators and resource managers in future risk assessments

## Materials and Methods

### Approach and experimental design

This study describes a series of outdoor open tank experiments to measure the degradation of herbicides under conditions more natural than those applied in standard flask tests. These tests were conducted in large open tanks with water circulation over the course of a year under both fully dark and light conditions (partially shaded, natural diurnal cycle) and in the presence and absence of natural sediments (Table 1).

**Table 1:** Four experimental treatments in the 40-tank open tank experiment. The PSII mix comprised of diuron, atrazine, hexazinone and tebuthiuron and the non-PSII mix of 2,4-D and metolachlor, each added at ~10 μg l^-1^. Each tank contained 120 l coastal seawater, temperature range (21–37°C).

Light conditions	Sediment conditions	
	Sediment free	Coastal Sediments
Dark	No herbicides (n = 3) PSII mix (n = 4) non-PSII mix (n = 3)	No herbicides (n = 3) PSII mix (n = 4) non-PSII mix (n = 3)
Light	No herbicides (n = 3) PSII mix (n = 4) non-PSII mix (n = 3)	No herbicides (n = 3) PSII mix (n = 4) non-PSII mix (n = 3)

The open fibreglass tanks (120 l) were situated in an outdoor glasshouse in two stacked rows of 10 (20 in the top rows and 20 in the bottom rows, each tank was 44 x 70 x 40 cm (W x L x D). The top 20 tanks were partially shaded (70%) and exposed to a natural diurnal cycle of moderate intensity up to a daily maximum of 700 μmol photons m^-2^s^-1^ (photosynthetically active radiation) as measured using light and temperature loggers placed at the base of tanks (Hobo UA-001-64, Onset, Bourne, MA). No UV penetration into the tanks was observed over the course of the experiment (Solartech UV Radiometer). The bottom row was fully shaded at all times. Evaporation was minimised with loose-fitting clear acrylic lids on the top row and opaque foam on the bottom row and water continuously circulated in each tank using aquarium circulation pumps (Turbelle Nanostream 6045; 4500 l/hour). While there was some visible turbidity generated for the first day of the experiment, this was not apparent during the remainder of the experiments. After every sampling period, evaporation losses were replenished with equal volumes of MilliQ freshwater. Logged temperatures averaged 28°C (range 21–37°C) in the light and 26°C (21–32°C) in the dark, with pH ranging from 8.1–8.4, salinity 32–35 psu and dissolved oxygen remained over 7.5 mg l^-1^ in all treatments.

### Sediments and water

Coastal seawater was collected from the shoreline of Australian Institute of Marine Science (19°16’ S, 147° 03’ E), Cape Cleveland, QLD and filtered to 20 μm to remove medium-large plankton. Intertidal sediments, containing no detectable concentrations of herbicides (see Results) were collected from the intertidal zone of low tide from Cockle Bay, Magnetic Island, Queensland (19°10’ S, 146° 49’ E). Both water and sediments were collected under the permit G12/35236.1 issued by the Great Barrier Reef Marine Park Authority. The experimental water was sampled and analysed for nutrients as previously reported [26] (S1 Table). Nutrients in the seawater were typical of coastal GBR waters at the beginning of the experiment [30]. Water samples were also taken from each tank for nutrient analysis at the end of the experiment (365 d) but inadvertent spiking of the water samples with an internal herbicide standard in acetone meant that only a subset of nutrients (NH_4_, PO_4_, NO_2_+NO_3_, NO_2_) were available for comparison between treatment types (S1 Table). The mean sediment particle size was 734 μm and contained 3.3%, 0.06% and 0.009% total carbon, total organic carbon and total nitrogen, respectively. The sediments were prepared one week prior to use by sieving (> 2 mm removed), thorough mixing and conditioning in shallow trays placed in two 1000 l tanks with 20 μm filtered and aerated seawater. During this conditioning process, half of the sediment was held in a dark trough (dark treatment) and the other half was held in 70% shaded sunlight (light treatment). This conditioning process allowed for microbial community transition to final experimental conditions prior to the start of the experiment (sediments conditioned in the light were used in the light treatments and sediments conditioned in the dark were used in dark treatments). To allow for periodic sediment sampling without disruption of sediment communities, the sediments were distributed into a single large and 11 small dishes in each tank which could be removed without disturbing the majority of the sediment. The large dishes (25 cm x 22 cm x 5 cm) were filled with 3.0 kg of sediment (wet weight) and the small ceramic dishes (6.5 cm diameter) with 70 g sediment. In total the sediments covered approximately 30% of the floor of each tank. Physical and chemical information on the seawater and sediments from the open tank experiment may be found in the Supplemental Information (S1 Table).

### Herbicide addition, sampling and analysis

Herbicide treatments and replicates (n = 3 or 4, see Table 1) were randomised among tanks. The six herbicides (Table 1) were purchased from Sigma Aldrich (>95% purity) and introduced as mixtures (using ethanol as a carrier < 0.03% v/v) to the seawater of each tank as per [26] at moderate concentrations (nominal 10 μg l^-1^, measured concentrations reported in Results) to maximise environmental relevance [6, 26, 28]. Herbicide concentrations can be detected in the nearshore marine environment at concentrations up to ~2 μg l^-1^ [31] and over 20 μg l^-1^ in heavily polluted rivers and estuaries [32]. The concentrations applied allowed direct analysis without pre-concentrations steps to maximise measurement precision. It is possible that isolated herbicides may degrade differently than those in mixtures; however, herbicides are almost always detected in mixtures in the environment [4, 31] and each contributed only ~1% of the overall dissolved organic carbon available for microbial degradation (S1 Table). Therefore, testing herbicide mixtures rather than single herbicides is environmentally relevant and enabled more frequent analyses as herbicides could be quantified simultaneously. Sample collection, internal standard addition and analytical techniques (HPLC-MS/MS using an AB/Sciex API5500Q mass spectrometer equipped with an electrospray interface and coupled to a Shimadzu Prominence HPLC system) were as previously reported [26]. Samples were collected on days 0, 21, 60, 100, 120, 180, 210, 240, 300 and 365. These 10 sampling points chosen included 60 d which is the standard duration of an OECD biodegradation test [28] and lasted for a year in recognition of the long persistence of these herbicides in standard flask tests [26]. Samples were run via direct injection, with a standard calibration at beginning and end, and additional quality control standards were run every 10 samples. Flow cytometry was used to quantify the microbial populations in the seawater used in the experiment (S6 Table). Samples were fixed with 5% formaldehyde and stored at 4°C. Sub-samples were stained using Sybr Green, diluted to 1:10,000, and allowed to develop in the dark for 30 min. Samples were run using a BD Accuri C6 cytometer (BD Biosciences, CA, USA) equipped with a red and blue laser (488 nm, 50mW maximum solid state; 640 nm, 30mW diode) and standard filter setup. Flow rate was 14 μl min^−1^, 10-μm core.

Sediments were sampled by removing small dishes using long aluminium tongs. The sediment samples were homogenised and then transferred to centrifuge tubes and weighed. The samples were centrifuged for 10 min at 3000 x *g*, excess water removed, and stored at -20°C prior to herbicide extraction [33]. Sediments were exhaustively extracted with acetone and concentrated to near dryness. Agilent QuEChERS kits and protocols were used for extract clean-up as per the manufacturer’s protocols (Agilent application notes 5990-3937EN and 5989-8614EN). Samples for 2,4-D required a pre-extraction hydrolysis step with 5M NaOH for 30 min [34]. The pH of the samples was then neutralised with 5N H_2_SO_4_. Cleaned extracts were evaporated to dryness and reconstituted into MilliQ water prior to LC/MS analysis [26]. Percent recovery for herbicide concentrations in the sediment samples were 87.1 to 118.5% (S2 Table).

### Data analysis

Half-life (*t*_1/2_) calculations assumed first order kinetics [26] and were estimated from the decline in experiment concentration of herbicide in seawater using the rate constant (*k*) slope of the data obtained from plots of the natural logarithm of the concentrations versus time (*t*), where *t*_1/2_ = ln(2)/*k* [35, 36]. Herbicide concentrations below the reporting limit were removed from the analysis [26]. The concentration data was tested for normality and analysed by repeated measures analysis of variance (ANOVA) using Number Cruncher Statistical System (NCSS 9) (Statistical and Power Analysis Software) across the time points sampled. Significance was determined if the resulting p-value was <0.05 between the initial concentration at day 0 and specified time point (e.g. day 60, day 365). The probability that *t*_1/2_ was statistically different between light and sediment treatments was tested by applying the *F* test (Graph Pad Prism V 6.0). Differences in *t*_1/2_ were considered significant when *p* < 0.05.

## Results

### 1. Overview

**Fig 1:**
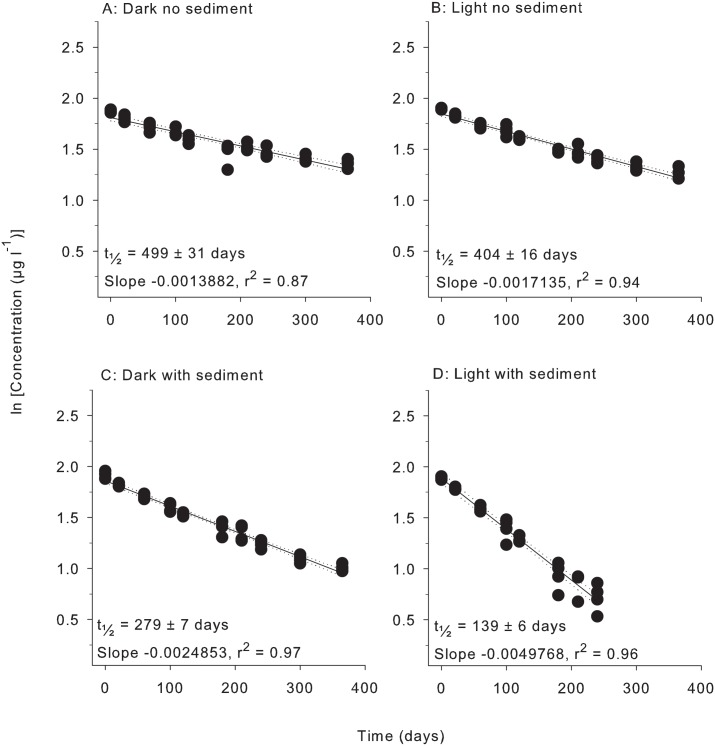
Experiment half-life results for diuron. ln(x) concentration of individual herbicide in PSII mixture for treatments: (A) Dark no sediment, (B) Light no sediment, (C) Dark with sediment, and (D) Light with sediment sampled up to 10 times over 365 days. Dashed lines represent 95% confidence intervals. Half-life reported ± SE.

**Fig 2:**
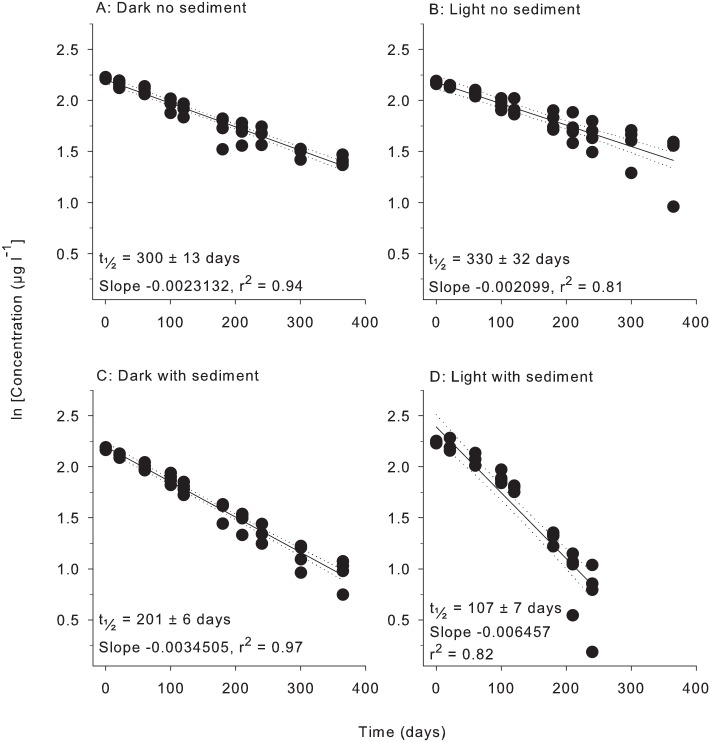
Experiment half-life results for atrazine. ln(x) concentration of individual herbicide in PSII mixture for treatments: (A) Dark no sediment, (B) Light no sediment, (C) Dark with sediment, and (D) Light with sediment sampled up to 10 times over 365 days. Dashed lines represent 95% confidence intervals. Half-life reported ± SE.

**Fig 3:**
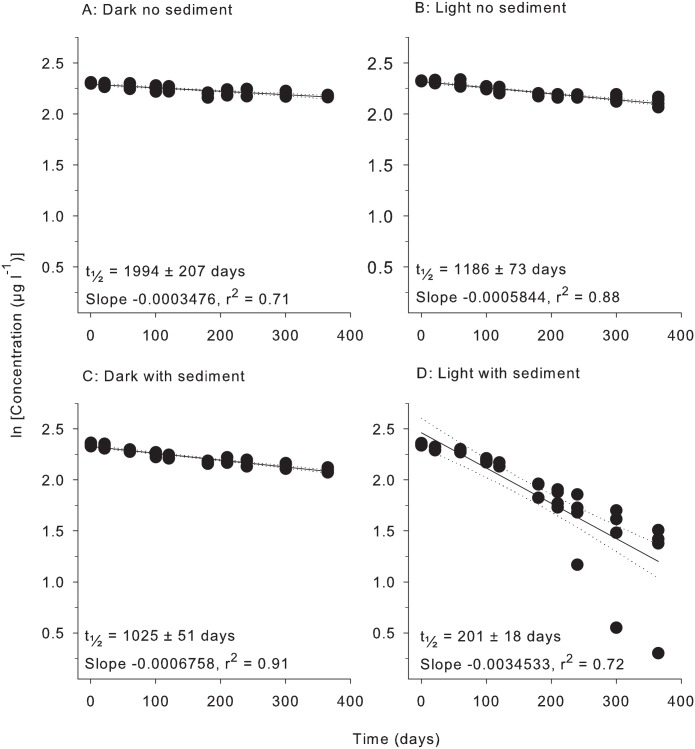
Experiment half-life results for hexazinone. ln(x) concentration of individual herbicide in PSII mixture for treatments: (A) Dark no sediment, (B) Light no sediment, (C) Dark with sediment, and (D) Light with sediment sampled up to 10 times over 365 days. Dashed lines represent 95% confidence intervals. Half-life reported ± SE.

**Fig 4:**
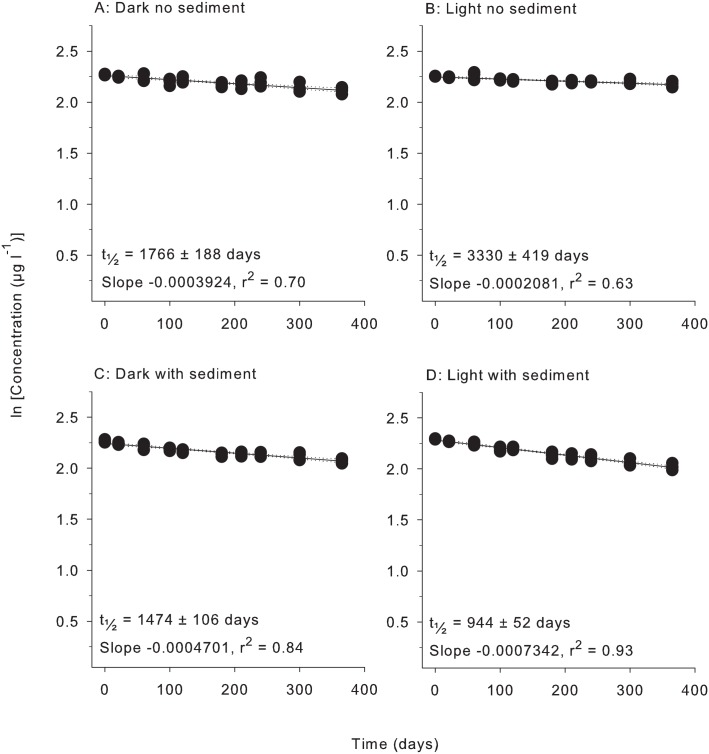
Experiment half-life results for tebuthiuron. ln(x) concentration of individual herbicide in PSII mixture for treatments: (A) Dark no sediment, (B) Light no sediment, (C) Dark with sediment, and (D) Light with sediment sampled up to 10 times over 365 days. Dashed lines represent 95% confidence intervals. Half-life reported ± SE.

**Fig 5:**
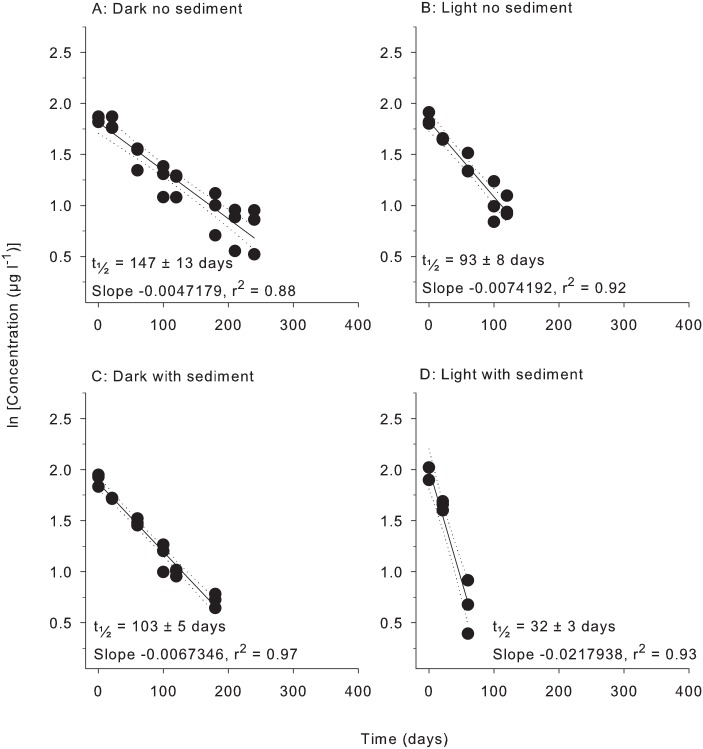
Experiment half-life results for metolachlor. ln(x) concentration of individual herbicide in non-PSII mixture for treatments: (A) Dark no sediment, (B) Light no sediment, (C) Dark with sediment, and (D) Light with sediment sampled up to 10 times over 365 days. Dashed lines represent 95% confidence intervals. Half-life reported ± SE.

**Fig 6:**
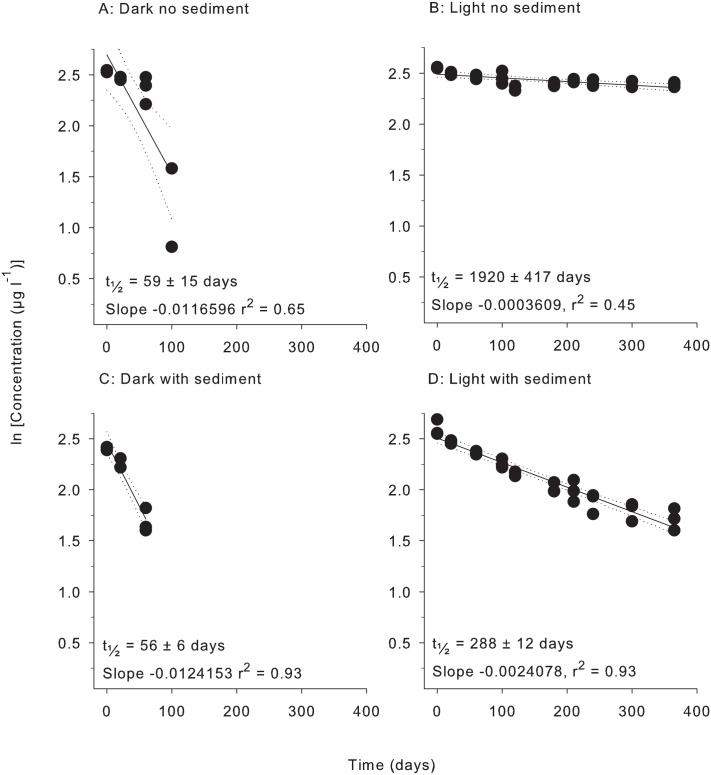
Experiment half-life results for 2,4-D. ln(x) concentration of individual herbicide in non-PSII mixture for treatments: (A) Dark no sediment, (B) Light no sediment, (C) Dark with sediment, and (D) Light with sediment sampled up to 10 times over 365 days. Dashed lines represent 95% confidence intervals. Half-life reported ± SE.

All herbicides degraded according to pseudo-first order kinetics with linear relationships evident for plots of ln (concentration) vs time under all four treatment conditions (Figs 1–6). All herbicides degraded significantly over 365 days in each of the treatment types (S3 Table) and the persistence for all conditions and herbicides calculated from Figs 1–6 are summarised in Table 2.

**Table 2:** Experimental half-lives (days ± SE). SE = Standard Error. The superscripts a,b,c,d represent significantly different slopes in Figs 1–6 (*F* test in Graph Pad Prism V 6.0, S5 Table), indicating differences in persistence between treatments for that herbicide.

Herbicide	Dark no sediment	Light no sediment	Dark with sediment	Light with sediment
Diuron	499 ± 31 ^a^	404 ± 16 ^b^	279 ± 7 ^c^	139 ± 6 ^d^
Atrazine	300 ± 13 ^a^	330 ± 32 ^a^	201 ± 6 ^b^	107 ± 7 ^c^
Hexazinone	1994 ± 207 ^a^	1186 ± 73 ^b^	1025 ± 51 ^b^	201 ± 18 ^c^
Tebuthiuron	1766 ± 188 ^a^	3330 ± 419 ^b^	1474 ± 106 ^a^	944 ± 52 ^c^
Metolachlor	147 ± 13 ^a^	93 ± 8 ^b^	103 ± 5 ^b^	32 ± 3 ^c^
2,4-D	59 ± 15 ^a^	1920 ± 417 ^b^	56 ± 6 ^a^	288 ± 12 ^c^

### 2. Degradation rates in the dark without sediments

Atrazine and diuron degraded 3- to 6–fold more rapidly than hexazinone and tebuthiuron in the dark without sediments (Table 2). The non-PSII herbicides metolachlor and 2,4-D degraded more rapidly than the PSII herbicides under these conditions with the shortest half-life being 59 days for 2,4-D (Table 2).

### 3. Light and sediment effects

The effects of light and sediment resulted in significantly different degradation rates (ln [concentration] versus time) for all herbicides under most experimental conditions (S3 Table). The persistence ratios (half-life of herbicide from a given treatment divided by the half–life of the same herbicide under control (dark, without sediments) conditions) demonstrates the scale of effect of the different treatments (Table 3). For example, the t_½_ for diuron of 404 d in the light, no sediments (Table 2) was shorter than under “standard” (dark, no sediment) conditions and consequently had a persistence ratio of 0.81 (Table 3). Light also significantly reduced the persistence of hexazinone and metolachlor, while atrazine degradation was not affected (Table 3). The presence of light increased the persistence of tebuthiuron almost 2-fold and 2,4-D over 30-fold to 1920 d (Tables 2 and 3). The addition of sediments under dark conditions increased the rates of degradation of diuron, atrazine, hexazinone and metolachlor by between (~30% and 50%) but had no impact on the degradation rates of tebuthiuron or 2,4-D (Table 2). The simultaneous effects of sediments and light resulted in the most rapid degradation of all herbicides except for 2,4-D (Tables 2 and 3). Atrazine exhibited the most rapid degradation of all the PSII herbicides under these conditions with a t_½_ of 107 d (Table 2), while the t_½_ of 32 d for metolachlor represented the most rapid degradation in the experiment. 2,4-D degraded almost 4-fold slower under these conditions than in the dark without sediments (Tables 2 and 3).

**Table 3:** The persistence ratio of half-lives between each treatment relative to “control” (dark, no sediment) conditions. The superscripts a,b,c,d represent significantly different ratios as calculated for and described in Table 2.

Herbicide	Dark no sediment	Light no sediment	Dark with sediment	Light with sediment
Diuron	1.00 ^a^	0.81 ^b^	0.56 ^c^	0.28 ^d^
Atrazine	1.00 ^a^	1.1 ^a^	0.67 ^b^	0.36 ^c^
Hexazinone	1.00 ^a^	0.59 ^b^	0.51 ^b^	0.10 ^c^
Tebuthiuron	1.00 ^a^	1.89 ^b^	0.83 ^a^	0.53 ^c^
Metolachlor	1.00 ^a^	0.63 ^b^	0.70 ^b^	0.22 ^c^
2,4-D	1.00 ^a^	33 ^b^	0.95 ^a^	4.9 ^c^

### 4. Metabolites

**Fig 7:**
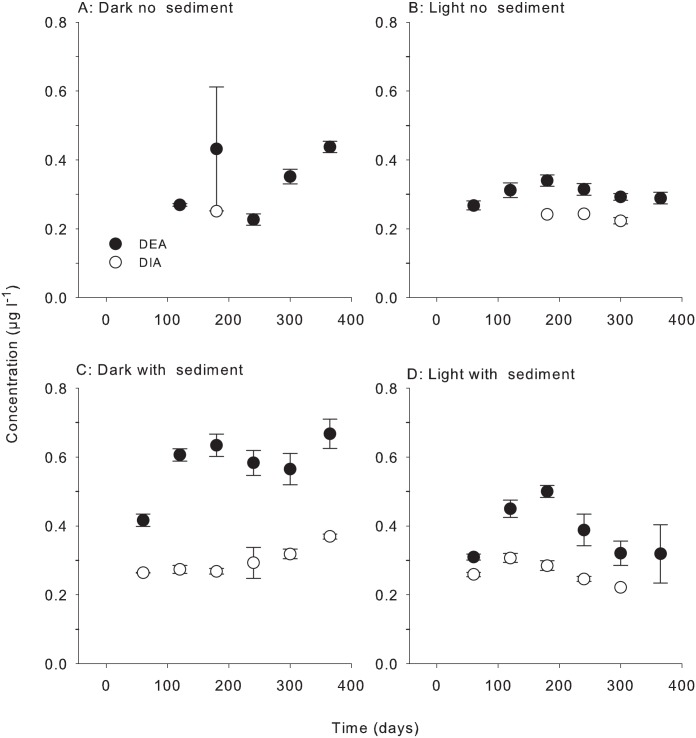
Concentration of metabolites of atrazine: DEA and DIA. Concentration (μg l^-1^) of individual herbicide metabolite in herbicide PSII mixture for treatments: (A) Dark no sediment, (B) Light no sediment, (C) Dark with sediment, and (D) Light with sediment sampled up to 10 times over 365 days. Bars represent ± SE.

Metabolites for atrazine were observed for all treatments which contained atrazine at concentrations above the reporting limit of 0.2 μg l^-1^. On average, desethyl atrazine (DEA) was detected more often and at slightly higher concentrations than desisopropyl atrazine (DIA) in the PSII treatments (See Fig 7). The maximum individual concentrations for the metabolites were 0.38 and 0.76 μg l^-1^ for DIA atrazine and DEA, respectively (365 day samples, dark with sediment treatment). These metabolites were detectable from the 60 day sampling onwards and were generally detected for the rest of the experiment. As the concentrations were close to the reporting limit no quantitative comparisons have been made. The main stable metabolite for diuron, 3,4-dichloroaniline (3,4-DCA), was not detected in water samples. Only these three metabolites (DEA, DIA, 3,4-DCA) were analysed as standards were not available for quantification of other transformation products.

### 5. Herbicides in sediments

Herbicides were analysed in sediments at 60 and 365 d and for all herbicides less than 1% of the total herbicides in each tank was associated with the sediments (S4 Table). For all herbicides, concentrations were higher in the 60 day samples (maximum ≤ 4 μg kg^-1^) than the 365 day samples; the only exception was tebuthiuron in the light which did not change between sampling periods. Metabolites of atrazine were detected at low concentrations (≤ 0.03 μg kg^-1^) in sediments (S4 Table).

## Discussion

An important element in assessing environmental risks posed by pesticides in the environment is to measure their potential degradation rates [1]. Our previous standard flask experiments demonstrated very long persistence of herbicides in coastal seawater [26, 29]. Here, under more environmentally relevant conditions, we confirm the long persistence of PSII herbicides in seawater and demonstrate very strong influences of variable light in combination with the presence of coastal sediments, which can reduce persistence by up to 10-fold. Although the effects of these environmental factors on persistence was consistent for the PSII herbicides and the non-PSII herbicide metolachlor, light (in the absence of UV) had an opposite effect on the degradation of 2,4-D which exhibited a 5-fold longer half-life in the presence of light and sediment in comparison to the standard dark, no sediment conditions. These results help explain the year-round presence of PSII herbicides in tropical estuarine and marine systems and underscore the need for more realistic experimental data on pesticide persistence in sensitive marine habitats.

### Effects of light on persistence

The presence of variable light: (i) significantly shortened the persistence of diuron, hexazinone and metolachlor; (ii) had no effect on atrazine; and (iii) increased the half-lives of tebuthiuron and, more dramatically 2,4-D. Photolysis or photodegradation may have contributed in cases of more rapid degradation (by 19–41%); however, other studies have reported only minor contributions of hydrolysis and photodegradation of diuron [37, 38] and hexazinone [24]. Photolysis has been shown to contribute to more rapid degradation of atrazine [39] and 2,4-D in [40] in shallow, full sunlight experiments; however, UV is likely to be highly attenuated in turbid nearshore waters of the GBR [41] and UV light did not penetrate the tanks in our setup, thus UV exposure would not have contributed to the influence of light on persistence. We previously demonstrated that low light (40 μmol photons m^-2^s^-1^) can significantly affect degradation of PSII herbicides in seawater, possibly due to changes in microbial community structure affecting the biodegradation [26]. However, the influence of light on the rates of biodegradation is unlikely to be predictable and may increase or decrease biodegradation, depending on intensity, duration and the initial community composition. In the cases of tebuthiuron and 2,4-D which exhibited (1.9-fold– 33-fold) longer persistence, the presence of light may have directly favoured bacteria less able to metabolise the herbicides (a direct effect) or may have changed the nature of other organic carbon in the system to form more metabolically available carbon sources (indirect effect). This could only be addressed by applying labelled substrates and herbicides and carefully assessing the nature and fate of transformation products [1], an exercise that could not be undertaken in our large experimental system and beyond the scope of the current study. The treatments exposed to light were slightly (mean 2°C) warmer than the fully dark treatments but, given our previous flask experiment [26] demonstrated only minor effects on persistence of a larger 6°C increase, temperature was unlikely to have had a major influence on persistence here. The herbicide concentrations applied in the current study were high enough to affect photosynthesis and growth in microalgae [42]; however, a recent study indicated no significant impact of similar concentrations of diuron on the community structure of bacteria, which are likely to have a greater direct impact on herbicide degradation [43]. Algal growth was noted but not quantified in all of the light treatments, indicating the herbicide concentrations were not toxic to all phototrophs.

### Effects of sediment on persistence

The presence of coastal sediments increased degradation of all herbicides in the water column (apart from 2,4-D) in comparison with standard (dark, no sediment) treatments. Less than 1% of the mass balances of herbicides were detected in the sediments, indicating the sediments were not an important “sink” for herbicides in the water column. Instead, the likely mechanism for increased degradation rates of 17–44% was due to differences in the microbial communities, including the possibility that coastal sediments in the tanks introduced a more natural, wider diversity, including more taxa capable of utilising the herbicides as a substrate. Although not quantified, the sediment-associated communities may have “seeded” the water column with taxa capable of degrading dissolved herbicides. Previous studies in freshwater have demonstrated more rapid (~3-fold) loss of atrazine and metolachlor in the presence of natural sediments, attributing this to more rapid biodegradation as well as sequestration by the sediments [44]. Up to 10% of the herbicides were physically associated with sediments in that study, highlighting differences in the potential of sediments (affected by type) on removal of herbicides from the water column and their potential bioavailability. The inclusion of sterile sediment treatments allowed Rice et al., [44] to postulate that the presence of sediments may also influence non-biotic degradation rates. For example, humic acids often associated with sediments have been shown to increase photolysis and hydrolysis of a range of contaminants [23, 45, 46]. Different sediment types (and levels) will contain different concentrations of minerals, nutrients and humic acids which may further aid non-biotic degradation processes and differentially sequester herbicides from the water column [44]. Atrazine and metolachlor had similar rates of degradation (224 and 98 days, respectively) in the absence of sediments in a microcosm experiment mimicking tropical freshwater wetland environments [47]. Again, the addition of natural sediments significantly increased removal from the water column, further reinforcing the utility of these more realistic conditions in experiments for predicting environmental persistence.

### The combined effects of light and sediments

The differences in degradation rates under the four experimental conditions highlights the strong effects of light and the presence of sediments on herbicide persistence in seawater. The most rapid degradation was observed for all herbicides (apart from 2,4-D) in the presence of both moderate light and coastal sediments (2–10- fold more rapid). Only the persistence of 2,4-D opposed this trend, being 5-fold more persistent in the absence of light and sediment. This effect could have been caused by the presence of different microbial communities (discussed below) and is consistent with longer persistence in the presence of low light observed in our standard flask experiments [26]. Conditions in the light + sediment treatment were the most environmentally relevant experimental degradation conditions applied and the herbicide half-lives obtained under these conditions represent the most reliable estimates for herbicide persistence in tropical marine waters available.

### Potential role of bacteria and nutrients

The long persistence of herbicides we report could be related to the limited capacity of natural microorganisms to metabolise the herbicides in the presence of other carbon sources and to the relatively low concentrations of the herbicides in solution. Thouland et al. [48], also described how the fate of chemicals in degradation studies are strongly influenced by the original inoculum used, largely depending on cell density, microbial diversity, and whether or not the microbial community may have been pre-exposed to the test chemical. Although the natural microbial populations used in the current experiment may have been previously exposed to low concentrations of these herbicides, in the tank experiments (as in the natural environment) they are likely to have access to a more abundant and diverse array of carbon sources (both dissolved in the seawater and associated with sediments) that may be more easily assimilated [49]. The nutrients in the tanks by the end of the experiment were similar to the relatively low concentrations reported for inshore waters of the GBR [30]. While the NH_4_, PO_4_ and NO_2_ concentrations were similar between the light and sediment treatments, NO_2_ + NO_3_ was 3–10-fold higher in the dark treatments, indicating impacts of light on nutrient cycling (e.g. NO_3_ concentrations can be influenced by microbial populations responding to light or can slowly degrade in the presence of light [50]) that may contribute to differences in microbial activity and potentially the more rapid degradation of 2,4-D in the dark. Future studies should specifically address the effects of nutrients on herbicide persistence in seawater. The active growth of bacterial communities in low-nutrient systems such as seawater is supported by the ability of most bacteria to adapt to a range of carbon sources [51]. However, the specific enzymatic pathways for metabolising complex organics like pesticides may only be induced above a “utilization threshold”, often in the range of 1–100 μg l^-1^ [51] and degradation may instead occur due to a slow, non-specific co-metabolism process [52]. The concentrations of herbicides applied in the current study were low in comparison to many studies and the microbial communities are less likely to induce herbicide metabolism of these low concentrations when other carbon sources are available. Concentrations of herbicides *in situ* are usually lower still (rarely above 10 μg l^-1^) underscoring the importance of degradation studies for risk assessment mimicking natural concentrations as closely as possible. The total number of water-borne bacteria was not affected by the presence of light or sediments (S6 Table); however, identifying the mechanistic pathways contributing to differences in persistence was beyond the scope of this study and could only be identified by: (i) quantifying all bacteria, including sediment-associated and biofilm bacteria; (ii) quantifying the genes related to the biodegradation pathways and (iii) a comprehensive analysis of transformation products [1].

### Environmental relevance

Despite the more rapid degradation observed for most herbicides in the presence of light and sediments, the half-lives of PSII herbicides were still > 100 days for diuron, atrazine and hexazinone, which helps explain their year-round presence in waterways of tropical Queensland [17]. The presence and concentration of these herbicides in the coastal zone is therefore more likely to be influenced by water exchange/dilution rather than degradation in the months following flood plumes. Even in the presence of light and sediments, the half-life of tebuthiuron was almost 3 years, indicating that this herbicide is very persistent in seawater. Tebuthiuron is commonly applied to control tree growth on grazing lands in the catchments of the southern GBR and its long persistence may contribute to detection in ~90% of water samples from this region [53]. The strong correlation of herbicides with freshwater plumes, which can extend for considerable distances along the coast, may limit the dilution of herbicides during the first weeks of a flood plume [16, 54]. Herbicide transport into the marine environment in river plumes also raises the question of relative persistence in fresh vs marine waters. The results from the present study indicate persistence of herbicides may be longer in seawater than freshwater [55, 56], but comparisons between studies are likely to be misleading as relative persistence has not been experimentally tested under similar conditions. Since herbicide degradation is largely dominated by biotic processes [1], the effects of light, sediments (including nutrients), temperature and salinity on degradation rates are highly likely due to influences on bacterial community structure and population densities [57].

Degradation was more rapid for all herbicides tested under these more environmentally relevant conditions than we previously reported from standard OECD- style flask experiments [26]. While the standard flask experiments provide repeatable conditions that enable reliable comparisons between laboratories and contaminants, half-lives from flask experiments may not be suitable for application in risk assessments. This is especially the case where unrealistically high concentrations of contaminant are tested in the presence of additional nutrients and inoculated with highly enriched and active microbial consortia (e.g. from wastewater plants and alike) [49]. Even when standard flasks studies are conducted to better simulate natural conditions they are often not performed for sufficient time to enable half-life calculations [26]. The static, yet open large tank experiments here, conducted in the presence of natural sediments and variable light and temperature provide conditions much more representative of those in the natural environment.

The current study helps address an important knowledge gap on pesticide transformation for regulators by measuring herbicide degradation at low concentrations in coastal seawater [1]. Future research needs to test the persistence of a wider range of pesticides at low concentrations in natural coastal waters of varying salinities, nutrients and in the presence of different sediment types. In particular, research into the mobility, transport, degradation and toxicity of newly registered or alternative herbicides is lacking and risks posed by these herbicides to the marine environment remains unclear [58]. Attention to the metabolites (transformation products) of herbicide degradation, which may be persistent and toxic, is also needed [1]. Herbicide metabolites were not specifically addressed in the current study due to the lack of available standards for quantification, although the presence of DEA and DIA confirmed the role of bacteria in the degradation of atrazine [37, 59]. In combination, new advances in LC-MS techniques and quantitative molecular tools should enable far more comprehensive analysis of transformation products, degradation pathways and potential in the laboratory and *in situ*.

## Supporting Information

S1 Table Nutrient measurements in seawater at the beginning and end of each treatment type. The seawater samples at the end of the experiment were spiked with an internal herbicide standard in acetone so only a subset of nutrients was available for comparison between treatment types. Nutrients were considered different between treatments when the one-way AONVA p < 0.05 and the superscripts a, b and c signify different nutrient concentrations between treatments.
(DOCX)

S2 Table Recovery of analytes in sediment extraction using Agilent Quenchers. n = 3.
(DOCX)

S3 Table Repeated measures ANOVA testing significance of degradation over time. (DOCX)

S4 Table Herbicide concentrations in sediments (μg kg-1). PSII herbicides n = 4; Non-PSII herbicides n = 3.
(DOCX)

S5 Table Results of statistical testing: Two- tailed test for differences between slopes (k). (DOCX)

S6 Table Flow cytometry bacterial counts at Time 0 and Time 365. Control n = 4; PSII herbicides n = 4; Non-PSII mixture n = 3.
(DOCX)
