# Supplementary material for: Degradation of Herbicides in the Tropical Marine Environment: Influence of Light and Sediment
Source: PLoS One. 2016 Nov 2;11(11):e0165890. doi: 10.1371/journal.pone.0165890 (PMC5091870; doi:10.1371/journal.pone.0165890)
Supplement: S1 Table — The seawater samples at the end of the experiment were spiked with an internal herbicide standard in acetone so only a subset of nutrients was available for comparison between treatment types. Nutrients were considered different between treatments when the one-way AONVA p < 0.05 and the superscripts a, b and c signify different nutrient concentrations between treatments. (DOCX) [file pone.0165890.s001.docx]

S1 Table: Nutrient measurements in seawater at the beginning and end of each treatment type. The seawater samples at the end of the experiment were spiked with an internal herbicide standard in acetone so only a subset of nutrients was available for comparison between treatment types. Nutrients were considered different between treatments when the one-way AONVA p < 0.05 and the superscripts a, b and c signify different nutrient concentrations between treatments.

| Parameter | Start of experiment | End of experiment | | | | |
| --- | --- | --- | --- | --- | --- | --- |
|  | All treatments | Dark-Sed | Dark+Sed | Light-Sed | Light+Sed | One-way ANOVA |
| Number of replicate tanks | 1 | 10 | 10 | 10 | 10 |  |
| DOC (mg l^-1^) | 0.57 | - | - | - | - |  |
| TOC (mg l^-1^) | 1.39 | - | - | - | - |  |
| NH_4_ (µmol l^-1^) | 0.46 | 0.75 (0.47) | 0.38 (0.06) | 0.66 (0.56) | 1.09 (0.77) | F_3_ = 2.73, p = 0.06 |
| PO_4_  (µmol l^-1^) | 0.17 | 0.15^a^ (0.03) | 0.18^a,b^ (0.01) | 0.14^a^ (0.02) | 0.22^b^ (0.05) | F_3_ = 3.46, p = 0.02 |
| NO_2_ + NO_3_  (µmol l^-1^) | 1.3 | 2.15^a^ (1.06) | 6.24^b^ (0.36) | 0.64^c^ (0.09) | 0.51^c^ (0.06) | F_3_ = 50.5, p < 0.01 |
| NO_2_  (µmol l^-1^) | 0.09 | 0.16^a^ (0.06) | 0.12^ac^ (0.01) | 0.08^c^ (0.01) | 0.08^c^ (0.01) | F_3_ = 3.67, p = 0.02 |
| TDP (µmol l^-1^) | 0.26 | - | - | - | - |  |
| TDN (µmol l^-1^) | 9.6 | - | - | - | - |  |
