# Supplementary material for: Degradation of Herbicides in the Tropical Marine Environment: Influence of Light and Sediment
Source: PLoS One. 2016 Nov 2;11(11):e0165890. doi: 10.1371/journal.pone.0165890 (PMC5091870; doi:10.1371/journal.pone.0165890)
Supplement: S2 Table — n = 3. (DOCX) [file pone.0165890.s002.docx]

S2 Table: Recovery of analytes in sediment extraction using Agilent Quenchers. n = 3.

| Herbicide | Average recovery ± SE |
| --- | --- |
| Diuron | 101.7 ± 6.1 |
| Atrazine | 87.1 ± 13.2 |
| Hexazinone | 118.5 ± 6.7 |
| Tebuthiuron | 99.5 ± 6.0 |
| Metolachlor | 113.9 ± 12.1 |
| 2,4-D | 96.3 ± 7.6 |
