# Supplementary material for: Degradation of Herbicides in the Tropical Marine Environment: Influence of Light and Sediment
Source: PLoS One. 2016 Nov 2;11(11):e0165890. doi: 10.1371/journal.pone.0165890 (PMC5091870; doi:10.1371/journal.pone.0165890)
Supplement: S3 Table — (DOCX) [file pone.0165890.s003.docx]

S3 Table: Repeated measures ANOVA testing significance of degradation over time.

| Dark no sediment | DF | f-ratio | p |
| --- | --- | --- | --- |
| Diuron | F_3,40_ | 62.21 | p<0.0001 |
| Atrazine | F_3,40_ | 135.82 | p<0.0001 |
| Hexazinone | F_3,40_ | 24.43 | p<0.0001 |
| Tebuthiuron | F_3,40_ | 24.81 | p<0.0001 |
| Metolachlor | F_2,30_ | 179.83 | p<0.0001 |
| 2,4-D | F_2,30_ | 154.73 | p<0.0001 |
|  |  |  |  |
| Light no sediment | DF | f-ratio | p |
| Diuron | F_3,40_ | 248.57 | p<0.0001 |
| Atrazine | F_3,40_ | 53.92 | p<0.0001 |
| Hexazinone | F_3,40_ | 61.53 | p<0.0001 |
| Tebuthiuron | F_3,40_ | 13.46 | p<0.0001 |
| Metolachlor | F_2,30_ | 268.37 | p<0.0001 |
| 2,4-D | F_2,30_ | 11.58 | p<0.0001 |
|  |  |  |  |
| Dark with sediment | DF | f-ratio | p |
| Diuron | F_3,40_ | 258.85 | p<0.0001 |
| Atrazine | F_3,40_ | 368.82 | p<0.0001 |
| Hexazinone | F_3,40_ | 75.97 | p<0.0001 |
| Tebuthiuron | F_3,40_ | 42.21 | p<0.0001 |
| Metolachlor | F_2,30_ | 415.42 | p<0.0001 |
| 2,4-D | F_2,30_ | 858.74 | p<0.0001 |
|  |  |  |  |
| Light with sediment | DF | f-ratio | p |
| Diuron | F_2,30_* | 994.57 | p<0.0001 |
| Atrazine | F_2,30_* | 296.14 | p<0.0001 |
| Hexazinone | F_2,30_* | 34.03 | p<0.0001 |
| Tebuthiuron | F_2,30_* | 87.02 | p<0.0001 |
| Metolachlor | F_2,30_ | 328.3 | p<0.0001 |
| 2,4-D | F_2,30_ | 75.27 | p<0.0001 |

* Lost replicate 4 for time point 120 d, Repeated ANOVA run with replicates 1-3 only.
