# Supplementary material for: Degradation of Herbicides in the Tropical Marine Environment: Influence of Light and Sediment
Source: PLoS One. 2016 Nov 2;11(11):e0165890. doi: 10.1371/journal.pone.0165890 (PMC5091870; doi:10.1371/journal.pone.0165890)
Supplement: S4 Table — PSII herbicides n = 4; Non-PSII herbicides n = 3. (DOCX) [file pone.0165890.s004.docx]

S4 Table: Herbicide concentrations in sediments (µg kg^-1^). PSII herbicides n = 4; Non-PSII herbicides n = 3.

| Herbicide |  | Sediment Dark | Sediment Light |
| --- | --- | --- | --- |
|  | Time | Average | Average |
| Diuron | 60 | 1.77 ± 0.08 | 1.65 ± 0.12 |
| Diuron | 365 | 1.28 ± 0.09 | 0.57 ± 0.12 |
| Atrazine | 60 | 0.59 ± 0.06 | 0.55 ± 0.05 |
| Atrazine | 365 | 0.18 ± 0.02 | 0.03 ± 0.01 |
| Desisopropyl Atrazine | 60 | 0.01 ± 0.01 | 0.01 ± 0.01 |
| Desisopropyl Atrazine | 365 | 0.01 ± 0.00 | 0.01 ± 0.00 |
| Desethyl Atrazine | 60 | 0.03 ± 0.00 | 0.03 ± 0.00 |
| Desethyl Atrazine | 365 | 0.03 ± 0.00 | 0.01 ± 0.00 |
| Hexazinone | 60 | 1.91 ± 0.13 | 1.66 ± 0.05 |
| Hexazinone | 365 | 1.03 ± 0.19 | 1.12 ± 0.34 |
| Tebuthiuron | 60 | 1.87 ± 0.18 | 1.51 ± 0.08 |
| Tebuthiuron | 365 | 1.1 ± 0.21 | 1.54 ± 0.20 |
| Metolachlor | 60 | 0.67 ± 0.13 | 0.14 ± 0.03 |
| Metolachlor | 365 | 0.08 ± 0.01 | BDL* |
| 2,4-D | 60 | 0.52 ± 0.01 | 1.36 ± 0.29 |
| 2,4-D | 365 | 0.02 ± 0.0 | 0.46 ± 0.08 |

*BDL = Below detection limit
