# Supplementary material for: Degradation of Herbicides in the Tropical Marine Environment: Influence of Light and Sediment
Source: PLoS One. 2016 Nov 2;11(11):e0165890. doi: 10.1371/journal.pone.0165890 (PMC5091870; doi:10.1371/journal.pone.0165890)
Supplement: S5 Table — (DOCX) [file pone.0165890.s005.docx]

S5 Table: Results of statistical testing: Two- tailed test for differences between slopes (k).

|  | DF | F | p |
| --- | --- | --- | --- |
| Dark no sediment vs light no sediment |  |  |  |
| Diuron | 1,76 | 8.7593 | 0.004106 |
| Atrazine | 1,76 | 1.24927 | 0.2672 |
| Hexazinone | 1,76 | 22.017 | p<0.0001 |
| Tebuthiuron | 1.76 | 14.3101 | 0.0003071 |
| Metolachlor | 1,35 | 10.4329 | 0.002693 |
| 2,4-D | 1,37 | 59.6548 | p<0.0001 |
| Dark with sediment vs light with sediment |  |  |  |
| Diuron | 1,75 | 252.007 | p<0.0001 |
| Atrazine | 1,75 | 130.569 | p<0.0001 |
| Hexazinone | 1,75 | 63.3583 | p<0.0001 |
| Tebuthiuron | 1,75 | 32.245 | p<0.0001 |
| Metolachlor | 1,23 | 90.6487 | p<0.0001 |
| 2,4-D | 1,35 | 78.7164 | p<0.0001 |
| Dark no sediment vs dark with sediment |  |  |  |
| Diuron | 1,76 | 100.928 | p<0.0001 |
| Atrazine | 1,76 | 63.2743 | p<0.0001 |
| Hexazinone | 1,76 | 44.7124 | p<0.0001 |
| Tebuthiuron | 1,76 | 2.13348 | 0.1482 |
| Metolachlor | 1,38 | 12.5437 | 0.001071 |
| 2,4-D | 1,16 | 0.0334772 | 0.8571 |
| Light no sediment vs light with sediment |  |  |  |
| Diuron | 1,75 | 395.61 | p<0.0001 |
| Atrazine | 1,75 | 212.685 | p<0.0001 |
| Hexazinone | 1,75 | 67.5164 | p<0.0001 |
| Tebuthiuron | 1,75 | 162.894 | p<0.0001 |
| Metolachlor | 1,20 | 55.9023 | p<0.0001 |
| 2,4-D | 1,56 | 189.969 | p<0.0001 |
| Dark no sediment vs light with sediment |  |  |  |
| Diuron | 1.75 | 430.615 | p<0.0001 |
| Atrazine | 1,75 | 232.097 | p<0.0001 |
| Hexazinone | 1,75 | 79.1258 | p<0.0001 |
| Tebuthiuron | 1,75 | 42.4027 | p<0.0001 |
| Metolachlor | 1,29 | 66.0661 | p<0.0001 |
| 2,4-D | 1,37 | 36.0735 | p<0.0001 |
| Dark with sediment vs light no sediment |  |  |  |
| Diuron | 1,76 | 66.5587 | p<0.0001 |
| Atrazine | 1,76 | 47.5453 | p<0.0001 |
| Hexazinone | 1,76 | 3.46509 | 0.06654 |
| Tebuthiuron | 1,76 | 38.3567 | p<0.0001 |
| Metolachlor | 1,29 | 1.17974 | 0.2864 |
| 2,4-D | 1,35 | 222.252 | p<0.0001 |
