# Supplementary material for: Degradation of Herbicides in the Tropical Marine Environment: Influence of Light and Sediment
Source: PLoS One. 2016 Nov 2;11(11):e0165890. doi: 10.1371/journal.pone.0165890 (PMC5091870; doi:10.1371/journal.pone.0165890)
Supplement: S6 Table — Control n = 4; PSII herbicides n = 4; Non-PSII mixture n = 3. (DOCX) [file pone.0165890.s006.docx]

S6 Table: Flow cytometry bacterial counts at Time 0 and Time 365. Control n = 4; PSII herbicides n = 4; Non-PSII mixture n = 3.

| Treatment | Day | Total bacterial counts (x 10^6^) ± SE |
| --- | --- | --- |
| Control dark no sediment | 0 | 2.65 ± 0.01 |
| Control dark with sediment | 0 | 2.62 ± 0.01 |
| Control light no sediment | 0 | 2.56 ± 0.04 |
| Control light with sediment | 0 | 2.45 ± 0.01 |
| Control dark no sediment | 365 | 2.50 ± 0.01 |
| Control dark with sediment | 365 | 2.49 ± 0.01 |
| Control light no sediment | 365 | 2.58 ± 0.02 |
| Control light with sediment | 365 | 2.51 ± 0.01 |
| PSII herbicides dark no sediment | 365 | 2.31 ± 0.01 |
| PSII herbicides dark with sediment | 365 | 2.29 ± 0.02 |
| PSII herbicides light no sediment | 365 | 2.41 ± 0.07 |
| PSII herbicides light with sediment | 365 | 2.41 ± 0.01 |
| Non-PSII mixture dark no sediment | 365 | 2.22 ± 0.02 |
| Non-PSII mixture dark with sediment | 365 | 2.23 ± 0.01 |
| Non-PSII mixture light no sediment | 365 | 2.52 ± 0.1 |
| Non-PSII mixture light with sediment | 365 | 2.48 ± 0.01 |
